# Treatment of Ankylosing Spondylitis Patients with Cervical Spinal Injury with Anterior Single-Stage Fixation with Bone Cement Augmentation

**DOI:** 10.3390/jcm13113131

**Published:** 2024-05-27

**Authors:** Dávid Süvegh, Ádám Juhász, Réka Viola, Mohammad Walid Al-Smadi, Árpád Viola

**Affiliations:** 1 Department of Traumatology, Semmelweis University, Fiumei út 17., 1081 Budapest, Hungary; david.suvegh@gmail.com (D.S.); juhasz.adam.se@gmail.com (Á.J.); 2 Department of Psychiatry, Peterfy Sandor Hospital, 1076 Budapest, Hungary; pallareka1004@gmail.com; 3 Department of Neurosurgery and Neurotraumatology, Dr. Manninger Jenő National Traumatology Institution, 1081 Budapest, Hungary; smadi996@hotmail.co.uk

**Keywords:** ankylosing spondylitis, cervical spinal fractures, anterior single-stage fixation, cement augmentation, cervical vertebral cement augmentation, cement leakage, subaxial stabilization

## Abstract

**Background/Objectives:** Cervical spine fractures in ankylosing spondylitis (AS) are characterized as highly unstable fractures posing an elevated risk of neurological deficit and a significantly elevated mortality rate. This study assesses the efficacy and safety of single-stage plate stabilization with ventral cement augmentation in treating subaxial cervical spine fractures in patients with AS. **Methods:** Over 86 months, 38 patients diagnosed with AS received ventral plate stabilization with cement augmentation after suffering unstable subaxial cervical fractures. No additional dorsal stabilization was used in any of these surgeries. **Results:** There were no complications as a result of cement leakage. During the follow-up period, screw loosening and implant displacement were documented in two out of 38 cases. At the time of data analysis, 17 patients who had undergone treatment had died, representing 44.7% of the total cases. Seven patients died within 1 month, two patients died within 6 months, four patients died within 1 year, and four patients died after 1 year. **Conclusions:** Our study shows that a single-stage anterior screw and plate fixation of the cervical spine with cement augmentation could be a feasible and effective method to treat cervical spine fractures in patients with AS.

## 1. Introduction

Ankylosing spondylitis (AS), also known as Bechterew’s disease or Pierre-Marie disease, is a chronic inflammatory disease that mainly affects the spine, resulting in pain, stiffness, and subsequent fusion of the vertebrae into a ‘bamboo spine’. The disease’s degenerative nature can significantly reduce the patient’s quality of life and increase the risk of different spinal injuries, most commonly subaxial cervical spine fractures [1,2,3].

Acute spinal fractures in AS most commonly occur in the subaxial cervical spine [3]. Acute spinal fractures in AS are hard to manage due to their high instability, potentially causing a higher risk of neurological deficits (ranging from 29% to 91%) and a significantly elevated mortality rate of 35% [4,5,6,7]. These fractures often involve unstable three-column injuries due to the extensive structural remodeling of the vertebrae, which accounts for a significant proportion of these injuries [8].

The management of severe cervical spine fractures in patients with AS requires a high level of expertise due to multiple comorbidities that complicate surgical intervention. This patient subgroup exhibits a significantly high cardiovascular risk, which accounts for approximately 60% of mortality in those diagnosed with AS [9].

Contraindications to anesthesia, such as increased cardiac risk and other comorbidities, may limit the options for surgical management. In cases of sub-axial unstable fractures, the restoration of adequate structural stability often requires the use of combined anterior and posterior instrumentation due to the poor quality of the bone. However, compromise solutions, such as simple posterior fixation or multi-session treatment, may be necessary due to the reduced surgical load-bearing capacity [8].

The use of plate stabilization combined with cement augmentation has shown promise for restoring stability to the anterior column of the cervical spine. E. Bayley et al. [10] and Oppenlander et al. have presented cases demonstrating the efficacy of these methods, particularly in osteoporotic patients and those with poor bone quality [11]. In vitro studies further support that cement augmentation notably enhances screw stability during ventral fixation [12].

In designing our study, we opted to use the AO Spine Subaxial Injury Classification System for the morphological classification of fractures. This classification system is known to have excellent inter- and intra-observer reliability, with the encoding of injury types being transparent. In addition to the morphological classes (A—compression, B—tension-band injury, C—translational injury, and F—facet injury), the classification system distinguishes several different injury subclasses (A1, A2, B1, B2, etc.). This clear coding facilitates the classification of fractures with similar characteristics and the statistical evaluation of their clinical consequences in research [13].

In this study, we aimed to assess the effectiveness and safety of single-stage plate stabilization and ventral cement augmentation for managing subaxial cervical spine fractures in patients with AS. This approach focuses on restoring structural stability and focusing on the challenges posed by the modified biomechanics of AS-affected spines. Through a retrospective analysis of the surgical outcomes, this study seeks to establish procedural efficacy, evaluate the incidence of complications such as cement leakage, and study the long-term impact on patient morbidity and mortality. By integrating these findings, we aim to improve and elevate the surgical techniques for this high-risk patient population, enhancing surgical outcomes and patient quality of life.

## 2. Materials and Methods

This study adopted a single-stage plate stabilization approach with ventral cement augmentation to manage unstable subaxial cervical spine fractures in patients with AS, eschewing posterior fixation. From November 2016 to December 2023, a cohort of 38 patients, ranging in age from 52 to 87 and with a mean age of 70.2, underwent this procedure. All surgeries were performed by a single senior surgeon using the Smith–Robinson anterolateral approach while the patients were supine; no additional dorsal stabilization was applied [14].

**Figure 1:**
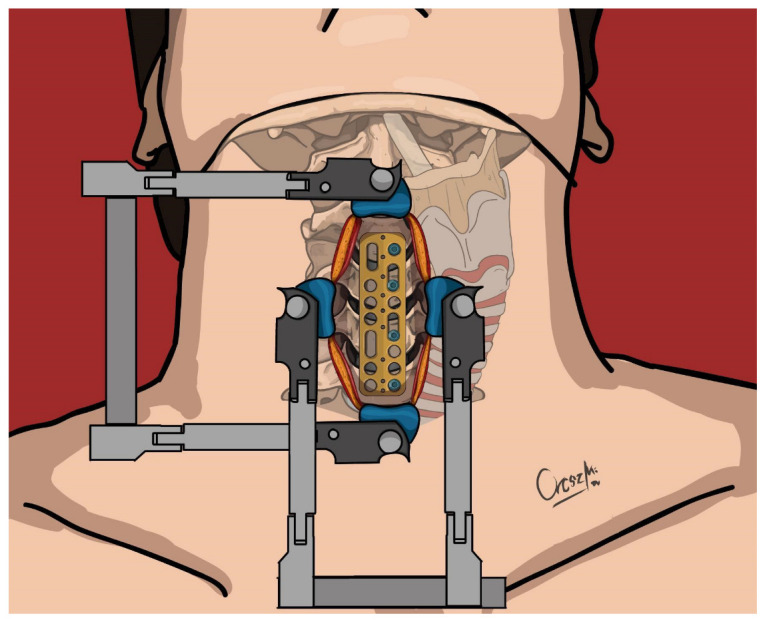
Positioning of the ventral cervical plate and insertion of screws to the left side—upper view.

**Figure 2:**
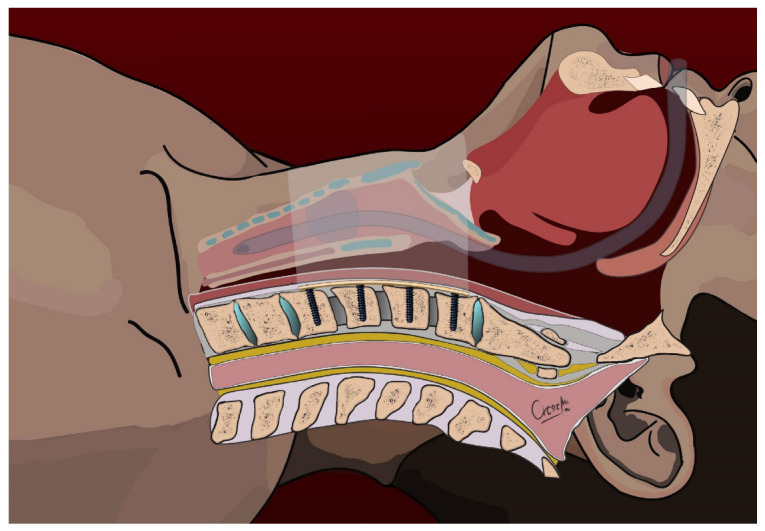
Positioning of the ventral cervical plate and insertion of screws to the left side—sagittal view.

The primary surgical intervention involved the placement of a cervical plate on the ventral surface of the spine (Caspar plate), which was secured with a screw per vertebra on the left side of the vertebral bodies using Caspar rescue screws (see Figure 1 and Figure 2). These were not augmented to minimize the risk of bone cement leakage, particularly in cases where the fracture lines involved the vertebral bodies (6 cases). In all cases, however, ventral stabilization aimed to connect two healthy vertebral bodies above and below the fracture. For the eight patients requiring discectomy due to calcified discs, Cervical Interbody Fusion Cages (CeSPACE^®^ PEEK, Aesculap, Hazelwood, MO, USA) were used to achieve fusion. Post-surgery, all patients were prescribed to wear a semi-rigid cervical collar for three months.

**Figure 3:**
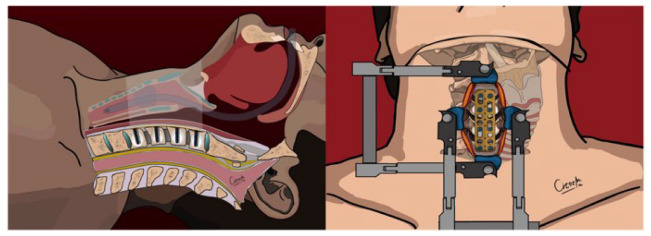
Cement augmentation through the right screwholes—upper and sagittal view.

During the procedure, a 10 G Vertebroplasty Needle (Diamond Tip, DePuy Synthes, Warsaw, IN, USA) was used to carefully insert Vertecem V+ polymethyl methacrylate (PMMA) into the vertebral bodies on the right side, aiming to inject a maximum of 1.5 mL of cement in a controlled manner under radiologic supervision with a C-arm (see Figure 3). In six cases, where the fracture lines involved one or more vertebral bodies, to minimize the risk of cement leakage, the involved vertebral bodies were not augmented.

**Figure 4:**
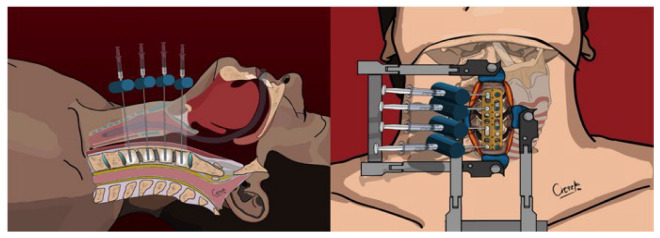
Final position of the cervical plate, screws, and cement—upper and sagittal view.

Intraoperative lateral x-ray fluoroscopy was utilized after each 0.2 mL of cement was injected to monitor for any signs of leakage. The injection was immediately halted if leakage was detected before reaching the intended volume of 1.5 mL. Following the cement injection, 4.5 mm screws (Caspar rescue screws) were placed monocortically to secure the right side of the plate, with screw lengths varying from 15 to 21 mm depending on the anatomical requirements (Figure 4).

The American Society of Anesthesiologists (ASA) physical status classification system was used to provide the patient’s physiological condition, aiding in the prediction of operative risks [15]. The patients’ cervical injuries were classified using the AO Spine Cervical Classification [16].

This study was conducted in strict adherence to the Declaration of Helsinki. It was approved by the Institutional Ethics Committee of Péterfy Sándor Street Hospital Outpatient and Accident Center (Registration number: 02/2021, dated 6 January 2021).

## 3. Results

From November 2016 to November 2023, we treated 38 patients with AS who suffered traumatic subaxial cervical spine injuries using plate fixation with cement augmentation via a ventral approach. The group had a mean age of 70.7 years (SD = 9.2 years) and consisted of 26 males (68.4%) and 12 females (31.6%). Regarding their general condition and anesthesia risk, the distribution was one patient in ASA I (2.6%), thirteen in ASA II (34.2%), twenty-two in ASA III (57.8%), and two in ASA IV (5.3%). (Table 1).

Concerning the mechanism of injury, 14 patients sustained injuries from traffic accidents (36.8%), 22 from low-energy trauma or falls (57.9%), and in two cases (5.3%), no specific traumatic event could be identified in their medical histories.

This study included five cases of revision surgery due to re-injury or screw loosening in previously untreated vertebral segments with cement augmentation (13.2%); the other 33 were primary. Using the AO Spine Cervical Classification, the most frequent are Type B3 (42.1%, *N* = 16) and Type C (34.2%, *N* = 13). Lesser occurrences of Types B1, B2, and F4 were also noted. Notably, one patient experienced both a B2 fracture at the C.IV.–C.V. vertebra and a B3 fracture at the C.VI.–C.VII. level. Almost all patients (94.7%; *N* = 36) had three-column lesions, except for two with injuries affecting the anterior two columns. Ten of the 38 patients (26.3%) exhibited some neurological deficits (Table 2).

Plate stabilization was extended to adjacent segments in 23 cases (60.5%). This was not required in five instances (13.2%), while in 10 other cases (26.3%), the extent of plate stabilization varied based on specific conditions. Cement was used to augment all segments in the stabilization in 32 cases (84.2%). Only the intact vertebrae were augmented in six cases with vertebral body fractures to mitigate the risk of cement leakage (15.8%). Postoperative imaging confirmed the correct placement of both plates and screws in all instances.

There were no complications from cement leakage. Postoperative CT scans categorized cement leakage into four groups: epidural to the spinal canal in 67.5% of cases (*N* = 27), near the vertebral artery in 22.5% (*N* = 9), into the neuroforamen in 7.5% (*N* = 3), and into the paravertebral space in 37.5% of cases (*N* = 15). In four cases (10.0%), no cement leakage was detected on the postoperative CT scans. During the follow-up period, screw loosening and implant displacement were documented in 2 of the 38 cases (Table 3).

During the follow-up period, screw loosening and implant displacement were documented in 2 out of 38 cases.

At the time of data analysis, 17 of the treated patients had died, representing 44.7% of the cases The minimum follow-up period was 91 days, while the maximum was 2674 days. The average survival of the deceased patients post-surgery was 264 days with seven patients dying within 1 month, two within 6 months, four within 1 year, and four after more than 1 year (Table 4).

## 4. Discussion

This study investigated the efficacy of single-stage plate stabilization combined with ventral cement augmentation for treating unstable subaxial cervical spine fractures in patients with AS.

Opting for a ventral approach without posterior fixation, this method was chosen considering this patient population’s comorbidities and complex surgical demands. The technique, including ventral cervical plate and cement augmentation, is designed to restore structural integrity. This method aligns with findings from prior studies that have demonstrated positive outcomes, with similar procedures aimed at stabilizing the anterior cervical column [10,11,17,18]. For instance, Waschke et al. treated nine patients with a standard one-level or two-level cervical corpectomy using an expandable titanium cage followed by anterior plating and vertebroplasty [18]. These techniques, which showed no instances of hardware failure, mirror our practice, where PMMA augmentation is applied through the screw pilot holes, similar to the method used by Oppenlander et al. There were no significant complications reported [11].

Our approach aimed to inject a maximum of 1.5 mL of cement per vertebral body under radiologic guidance using a C-arm, with the actual volume ranging from 0.8 to 1.5 mL. The strategy to augment only intact vertebrae adjacent to fractures minimized the risk of cement leakage. Cement-leakage assessments revealed no adverse events, with leakage patterns typically occurring in the epidural and paravertebral spaces.

Reyes-Soto et al. used PMMA-filled mesh after odontoidectomy to perform a transoral clivus-cervical stabilization in three cases. After removing the tumorous odontoid, they formed a PMMA-filled mesh to replace it and anchored the clivus-cervical plate to it, with promising results [19].

Bayley et al.’s treatment of seven patients with ventral vertebral augmentation introduced 0.2–0.25 mL of Kyphon cement into the screw holes prior to securing the anterior plate, also resulting in a complication-free seven-month follow-up period [10].

Oppenlander et al. presented promising results in their case study of an elderly osteoporotic patient [11]. After performing discectomies at the C3–C7 levels, they placed an anterior cervical plate with PMMA augmentation into the vertebral bodies using the screw pilot holes. Despite increasing the screw pullout strength, due to the grossly poor bone quality of the patients, a postoperative HALO was placed for three months, reaching full healing of the interbody fusions without screw pullout and implant displacement at 6-month follow-up.

The study encompassed 38 AS patients, predominantly male, with a mean age of 70.7 years and a high prevalence of comorbid conditions, as indicated by their ASA classifications. The injuries mainly resulted from low-energy trauma or falls, predominantly affecting the CIV–V and CV–VI levels. Notably, 26.3% of these patients experienced neurological deficits. Comparatively, Caron et al. found similar injury patterns in a group of 67 patients, with a high incidence of spinal cord injuries in cervical fractures primarily located at the C6–C7 levels [20].

Previous subaxial spine-injury classifications relied predominantly on anatomical descriptions and exhibited poor clinical utility and reliability. The introduction of the AO Spine Subaxial Injury Classification System represents a significant advancement, offering a concise and comprehensive framework for categorizing these injuries based on morphology and stability [13]. A validation study was conducted to underscore its rigor and comprehensiveness [21]. By inviting participation from a diverse group of AO Spine members across six geographic regions, this study ensures broad representation and increases the generalisability of its findings. Furthermore, the use of live webinar conferences and standardized training sessions prior to assessment minimizes potential biases and ensures consistent application of the classification system.

The results reveal high levels of agreement among validation members in classifying subaxial cervical spine injuries according to the AO Spine Subaxial Injury Classification System. The excellent interobserver reliability and intraobserver reproducibility observed for fracture morphology, subtype, and facet injury classification reaffirm the robustness of the classification system. These findings support its widespread applicability as a communication tool for describing subaxial cervical spine-injury patterns on a global scale.

The classification of fractures according to the AO Cervical Spine Classification showed a predominance of type B3 injuries and the majority of patients had three column lesions. Caron et al., using a different classification, found that the majority of injuries were extension injuries, which is similar to B3 fractures in the AO Spine Classification [13,20].

The postoperative mortality rate was 44.7%, with a one-year mortality of 34.2%. Comparatively, Caron et al. reported a one-year mortality of 32% in conservatively treated patients and 23% in those who underwent surgery, emphasizing that thoracolumbar fractures, which were included in their analysis, typically show lower mortality rates than cervical fractures [20].

The American Spinal Injury Association (ASIA) developed the American Spinal Injury Association Impairment Scale (AIS), the gold standard in evaluating spinal injuries. To better characterize the neurological deficit in spinal injury patients, it uses myotomal-based motor examination and dermatomal-based sensory examination. This standardized grading system helps physicians objectively assess the motor function of key muscle groups and sensory points, leading to better identification of the neurologic levels of injury, as well as recording changes in neurological status and improving research. As the AIS Scores were not recorded or available for every patient, despite how informative and useful it would be, unfortunately, this study does not report ASIA grading or AIS Scores [22].

Enhanced recovery after surgery (ERAS) protocols, which have been established in general surgery, have recently become increasingly popular in neurosurgical care. The essence of ERAS protocols is to establish pre-, intra-, and post-operative management approaches that use a multimodal strategy to reduce the stress associated with surgery in order to allow for faster recovery following major surgical procedures. ERAS protocols define preoperative, intraoperative, and postoperative recommendations. In the field of lumbar fusion, there are already guidelines with ERAS recommendations [23].

However, there is a lack of data available for cervical subaxial injuries. Our study may serve as a basis for future comprehensive work on ERAS recommendations for subaxial fractures in patients with degenerative spine disease [24].

Additionally, recent studies have emphasized the potential of innovative regenerative approaches for enhancing surgical outcomes for spinal injuries. Notably, Montemurro et al. highlight the efficacy of integrating autologous growth factors, platelet-rich plasma (PRP), and stem cells (SCs) with advanced surgical technologies, such as piezosurgery and lasers [25]. This integration has significantly improved the grade of surgery and implant procedures. Furthermore, the use of high-efficiency bio-scaffold solutions, such as Compact-bio BoneR, coupled with hormones and essential vitamins like D, C, and K, has been reported to induce a synergistic effect in bone regeneration, which is critical for patients with cervical spinal injuries due to AS.

This holistic approach underlines the importance of addressing immune–endocrine–metabolic conditions, which play a crucial role in the success of surgical interventions. Accurate pre-operative screening and managing the patient’s pre-existing conditions, as suggested in the review, could thus provide a valuable and feasible therapeutic strategy for enhancing bone-defect healing in the context of complex spine surgeries.

## 5. Conclusions

This comprehensive approach highlights the potential of ventral stabilization techniques for managing complex spinal injuries in a high-risk group, offering insights into procedural efficacy and patient outcomes.

While the study has its advantages, it is important to recognize its limitations, including its retrospective design and the relatively small cohort. These factors limit the generalizability of the findings. On the positive side, the research offers valuable insights into the treatment of subaxial cervical spine fractures in patients with AS. To build on this foundation, future studies could benefit from larger sample sizes, prospective methodologies, and the inclusion of control groups, which would help to further substantiate the efficacy and applicability of this surgical approach.

Our study shows that a single-stage anterior screw and plate fixation of the cervical spine with cement augmentation could be a feasible and effective method to treat cervical spine fractures in patients with AS. More studies are needed to better understand its benefits and limitations.

## Figures and Tables

**Table 1:** Patient demographics and clinical characteristics.

Variable	Value
Total Patients	38
Age Range	52–87 years
Median Age	71 years
Mean Age	70.2 years
Gender Distribution	26 males (68.4%), 12 females (31.6%)
ASA Classification	
- ASA I	1 patient (2.6%)
- ASA II	13 patients (34.2%)
- ASA III	22 patients (57.8%)
- ASA IV	2 patients (5.3%)
Mechanism of Injury	
- Traffic Accident	14 patients (36.8%)
- Low-energy Trauma or Falls	22 patients (57.9%)
- Unknown	2 patients (5.3%)
Type of Surgery	
- Primary	33 cases
- Revision	5 cases
Neurological Deficit	10 patients (26.3%)

**Table 2:** Injury classification and surgical details.

Injury Type	Number of Cases	Percentage
Type B3 Fractures	16	42.1%
Type C Fractures	13	34.2%
Other Fractures	9	23.7%
3-Column Lesions	36	94.7%
Anterior 2-Column	2	5.3%

**Table 3:** Surgical outcomes and complications.

Outcome	Details
Cement-Leakage Location	
- Epidural Spinal Canal	27 cases (67.5%)
- Vertebral Artery Area	9 cases (22.5%)
- Neuroforamen	3 cases (7.5%)
- Paravertebral Space	15 cases (37.5%)
No Leakage	4 cases (10.0%)
Screw Loosening	2 cases
Postoperative Mortality	17 deaths (44.7%)
Survival Time Post-Surgery	Average 264 days

**Table 4:** Follow-up and mortality rates.

Timeframe	Number of Deaths
Within 1 month	7
Within 6 months	2
Within 1 year	4
After 1 year	4

## Data Availability

The anonymized dataset on which the statistical analysis is based is not publicly available but will be provided upon request to the editorial board.
